# 3-Ketosteroid 9α-hydroxylase enzymes: Rieske non-heme monooxygenases essential for bacterial steroid degradation

**DOI:** 10.1007/s10482-014-0188-2

**Published:** 2014-05-21

**Authors:** Mirjan Petrusma, Robert van der Geize, Lubbert Dijkhuizen

**Affiliations:** Department of Microbiology, Groningen Biomolecular Sciences and Biotechnology Institute (GBB), University of Groningen, Nijenborgh 7, 9747 AG Groningen, The Netherlands

**Keywords:** 3-Ketosteroid 9α-hydroxylase, Rieske mono-oxygenase, 9α-Hydroxylation, *Rhodococcus*, *Mycobacterium*, Steroid biotransformation

## Abstract

Various micro-organisms are able to use sterols/steroids as carbon- and energy sources for growth. 3-Ketosteroid 9α-hydroxylase (KSH), a two component Rieske non-heme monooxygenase comprised of the oxygenase KshA and the reductase KshB, is a key-enzyme in bacterial steroid degradation. It initiates opening of the steroid polycyclic ring structure. The enzyme has industrial relevance in the synthesis of pharmaceutical steroids. Deletion of KSH activity in sterol degrading bacteria results in blockage of steroid ring opening and is used to produce valuable C19-steroids such as 4-androstene-3,17-dione and 1,4-androstadiene-3,17-dione. Interestingly, KSH activity is essential for the pathogenicity of *Mycobacterium tuberculosis*. Detailed information about KSH thus is of medical relevance, and KSH inhibitory compounds may find application in combatting tuberculosis. In recent years, the 3D structure of the KshA protein of *M. tuberculosis* H37Rv has been elucidated and various studies report biochemical characteristics and possible physiological roles of KSH. The current knowledge is reviewed here and forms a solid basis for further studies on this highly interesting enzyme. Future work may result in the construction of KSH mutants capable of production of specific bioactive steroids. Furthermore, KSH provides an promising target for drugs against the pathogenic agent *M. tuberculosis*.

## Introduction

**Fig. 1:**
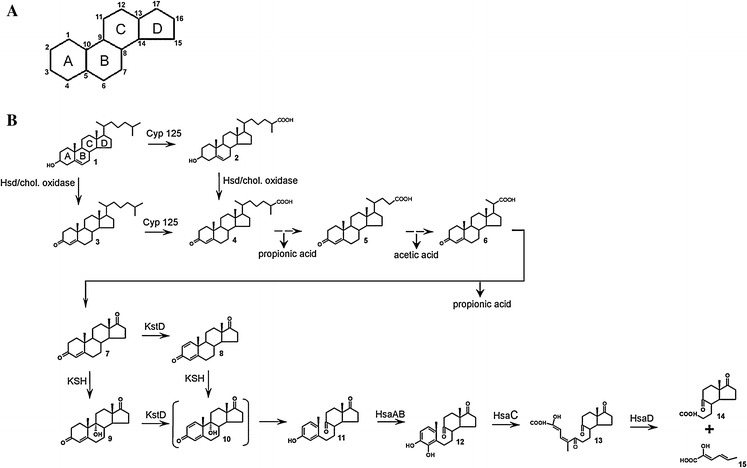
**a** The basic polycyclic ring structure of sterols and steroids with carbon atoms *1*–*17*. **b** Proposed cholesterol catabolism in *Rhodococcus* species and *Mycobacterium tuberculosis* (Adapted from Van der Geize et al. 2007). Dashed arrows indicate multiple enzymatic steps. The depicted steroids are *1* 5-cholestene-3β-ol (cholesterol), *2* 5-cholestene-26-oic acid-3β-ol, *3* 4-cholestene-3-one, *4* 4-cholestene-26oic acid-3-one, *5* 4-cholestene-24oic acid-3-one, *6* 3-oxo-23,24-bisnorchola-4-ene-22-oic acid, *7* 4-androstene-3,17-dione (AD), *8* 1,4-androstadiene-3,17-dione (ADD), *9* 9α-hydroxy-4-androstene-3,17-dione (9OHAD), *10* 9α-hydroxy-1,4-androstadiene-3,17-dione (ADD), *11* 3-hydroxy-9,10-secoandrost-1,3,5(10)-triene-9,17-dione (3-HSA), *12* 3,4-dihydroxy-9,10-secoandrost-1,3,5(10)-triene-9,17-dione (3,4-DHSA), *13* 4,5–9,10-diseco-3-hydroxy-5,9,17-trioxoandrosta-1(10),2-diene-4-oic acid (4,9-DSHA), *14* 9,17-dioxo-1,2,3,4,10,19-hexanorandrostan-5-oic acid (DOHNAA), *15* 2-hydroxyhexa-2,4-diene-oic acid (HHD). The compound between *brackets* is chemically unstable. *Hsd* 3β-hydroxy steroid dehydrogenase, *Cyp 125* cytochrome P450 CYP125, *KstD* 3-ketosteroid dehydrogenase, *KSH* 3-ketosteroid 9α-hydroxylase, *HsaAB* 3-hydroxy-9,10-seconandrost-1,3,5(10)-triene-9,17-dione 4-hydroxylase, *HsaC* 3,4-dihydroxy-9,10-secoandrost-1,3,5(10)-triene-9,17-dione dioxygenase, *HsaD* 4,5–9,10-diseco-3-hydroxy-5,9,17-trioxoandrosta-1(10),2-diene-4-oic acid hydrolase. The *A*, *B*, *C* and *D ring* of the steroid polycyclic ring structure are indicated in compound 1 (Petrusma 2011)

Early observations that microbes are able to degrade cholesterol were made by Söhngen (1913). Since then, a range of bacteria were found to be able to utilize sterols (Arima et al. 1969; Nagasawa et al. 1969), many of which are actinobacteria (e.g. *Rhodococcus*, *Mycobacterium*) (Donova 2007; Fernandes et al. 2003; Malaviya and Gomes 2008 (reviews)). Various enzyme steps in the bacterial sterol degradation pathways remain to be characterized, but in recent years knowledge on sterol degradation has greatly increased. A cholesterol catabolism gene cluster was identified in *R. jostii* RHA1 and a cholesterol degradation pathway was predicted (Van der Geize et al. 2007) (Fig. 1). Cholesterol is a C27 sterol and degradation of its side chain to a C22 intermediate occurs by a process similar to β-oxidation of fatty acids, yielding propionic acid and acetic acid. The subsequent formation of C19 steroids yields another propionic acid (Sih et al. 1968). In *M. tuberculosis*, metabolic labeling studies at carbon atom C26 showed that the propionic acid released from the side chain is a precursor in the production of phthiocerol dimycocerosate (PDIM), a surface lipid (Ouellet et al. 2010). The C19 central metabolite derived from cholesterol is AD, containing the polycyclic ring structure. Steroid ring degradation has been described in some detail for the Gram-negative bacterium *Comamonas testosteroni* TA441 (Horinouchi et al. 2012) and for the actinobacteria *Rhodococcus equi* (*Nocardia restrictus*) ATCC 14887 (Gibson et al. 1966; Sih et al. 1968), *R. jostii* RHA1 and *M. tuberculosis* (Van der Geize et al. 2007). Several enzymes have been characterized from these organisms as well as from *Rhodococcus erythropolis* SQ1 (e.g. Van der Geize et al. 2002), *Nocardia corralina* (e.g. Itagaki et al. 1990) and *R. rhodochrous* DSM43269 (e.g. Petrusma et al. 2009). Metabolic labelling studies at carbon atom C4 of cholesterol in *M. tuberculosis* revealed that this carbon atom in the ring structure is converted to CO_2_, suggesting the generation of energy via the tricarboxylic acid (TCA)-cycle (Pandey and Sassetti 2008). Opening of the steroid polycyclic ring structure in these organisms occurs via the so called 9,10-seco pathway. Recently, an alternative 2,3-seco pathway was proposed for aerobic sterol degradation, which does not use oxygenase enzymes for side chain cleavage and for the steroid ring opening event. In the latter case, cleavage of the steroid polycyclic ring structure starts with a hydrolytic attack on the A-ring. The proposed pathway is based on identification of cholesterol degradation intermediates in the gram negative bacterium *Sterolibacterium denitrificans* (Wang et al. 2013).

In actinobacteria, employing the 9,10-seco pathway, the steroid B-ring is opened by dehydrogenation of the steroid A-ring and by C9α-hydroxylation. The sequence of these reactions is not known. The dehydrogenation step is performed by 3-ketosteroid dehydrogenase enzymes (KSTD) (Itagaki et al. 1990; Knol et al. 2008; Van der Geize et al. 2000), whereas 3-ketosteroid 9α-hydroxylase enzymes (KSH) incorporate a hydroxyl moiety at C9 (Van der Geize et al. 2002). The resulting steroid structure, 9α-hydroxy-1,4-androstadiene-3,17-dione (9OHADD), is chemically unstable and spontaneously hydrolyzes to the phenol 3-hydroxy-9,10-secoandrost-1,3,5(10)-triene-9,17-dione (3-HSA). 3-HSA is further degraded by the HsaABCD enzymes. This involves C4-hydroxylation by the flavin-dependent monooxygenase HsaAB (Dresen et al. 2010), cleavage of the A-ring by the extradiol dioxygenase HsaC (Yam et al. 2009) and hydrolysis by HsaD (Lack et al. 2010) yielding 9,17-dioxo-1,2,3,4,10,19-hexanorandrostan-5-oic acid (DOHNAA) and 2-hydroxyhexa-2,4-diene-oic acid (HHD). The fate of the DOHNAA product is not entirely clear yet. HHD is further degraded to propionic acid and pyruvate. The two main events in microbial sterol catabolism are C17-side chain degradation and opening of the polycyclic ring structure. The sequence of these events may differ between organisms. *R. jostii* RHA1 is thought to first attack the sterol side chain followed by opening of the steroid ring structure (Rosłoniec et al. 2009). In *M. tuberculosis* H37Rv both events may occur simultaneously, since its KSH displays high substrate preference for a CoA thioester intermediate of cholesterol side chain degradation (Capyk et al. 2011). In bile acid transformations with three *Rhodococcus* strains, 9,10-secosteroid intermediates with partially degraded C17 side chains were detected, suggesting that C17-side chain degradation and opening of the steroid polycyclic ring structure occur simultaneously (Costa et al. 2013a, b).

Although microbial steroid hydroxylation is well documented, starting with the discovery of progesterone 11α-hydroxylation by a *Rhizopus* species in 1952 (Peterson and Murray 1952), the responsible enzymes are in most cases not known. Over the years, several cytochrome P450 (CYP) enzymes were found to be involved in steroid hydroxylation (Donova and Egorova 2012 (review); Fernandes et al. 2003 (review); Holland 1999 (review); Rosłoniec et al. 2009). Examples of microbial steroid hydroxylation mediated by P450 enzymes are 11α-hydroxylation by *Rhizopus nigricans* (Breskvar et al. 1991), 11β-hydroxylation by *Curvularia lunata* (Suzuki et al. 1993) and 14α-hydroxylation by *Mucor piriformis* (Madyastha and Joseph 1993) and the 26/27-hydroxylases CYP125 of *Rhodococcus jostii* RHA1 (Rosłoniec et al. 2009) and CYP142 of *Mycobacterium tuberculosis* (Driscoll et al. 2010).

Various steroid hydroxylases play a role in microbial steroid degradation, resulting in hydroxylation of steroids at virtually every carbon atom (Fernandes et al. 2003; Mahato and Garai 1997; Donova 2007; Donova and Egorova 2012 (reviews)). Pharmaceutical steroids have a wide range of applications and hydroxylated steroids, often displaying high bioactivity, are of large industrial and medical relevance. The pharmaceutically most interesting sites for hydroxylation are at carbon atoms C-7α, C-9α, C-11α, C-11β, C-16α, C-17α (Donova and Egorova 2012 (review)). Steroid C9-hydroxylation and C11-hydroxylation reactions for instance are important for the production of corticosteroids (Kano et al. 1979; Megges et al. 1990).

Steroid C9α-hydroxylation is catalyzed by the 3-ketosteroid 9α-hydroxylase (KSH) enzyme. KSH is a key-enzyme in bacterial steroid degradation and initiates opening of the steroid polycyclic ring structure. The two-component Rieske-type non-heme monooxygenase KSH consists of the terminal oxygenase KshA and the ferredoxin reductase KshB. Deletion of KshA activity in sterol degrading bacteria results in blockage of steroid ring opening and is used to produce valuable C19-steroids, such as 4-androstene-3,17-dione (AD) and 1,4-androstadiene-3,17-dione (ADD), core metabolites in the sterol degradation pathway (Andor et al. 2006; Van der Geize et al. 2002; Wilbrink et al. 2011). These steroids can be used as precursors for virtually all pharmaceutically interesting steroids (Wang et al. 2011 (review)). Interestingly, it has been reported that KSH is essential for the pathogenicity of *M. tuberculosis* (Hu et al. 2010). Insight into this important enzyme can provide new leads for the development of inhibitors to be used as drugs against this notorious pathogen.

## 3-Ketosteroid 9α hydroxylase, a multimeric two component Rieske type non-heme oxygenase

KSH is a key enzyme in bacterial sterol degradation, initiating the opening of the steroid polycyclic ring structure by C9α-hydroxylation (Gibson et al. 1966; Van der Geize et al. 2002). Characterization of enzymes involved in C9α-hydroxylation of steroids started with a partly purified NADH dependent three component enzyme system of *Nocardia* sp. M117 (Strijewski 1982). The enzymes were identified as a flavoprotein reductase and two iron–sulphur proteins. Genes encoding a two component KSH enzyme system were first identified in *R. erythropolis* SQ1 (Van der Geize et al. 2002). UV-mutagenesis of *R. erythropolis* SQ1 and screening and characterization of mutants impaired in growth on AD but still able to grow on 9α-hydroxyAD (9OHAD) revealed that the *kshA* and *kshB* genes are involved in steroid C9α-hydroxylation. Unmarked gene deletion in *R. erythropolis* SQ1 (van der Geize et al. 2001) of either *kshA* or *kshB* both resulted in mutant strains incapable of growth on AD. Based on its amino acid sequence, KSH was identified as a Rieske non-heme monooxygenase enzyme system. Monooxygenases are also called mixed-function oxygenases because they incorporate one oxygen atom into the substrate and one atom is reduced to H_2_O (Harayama et al. 1992; Mason and Cammack 1992 (reviews)).

**Fig. 2:**
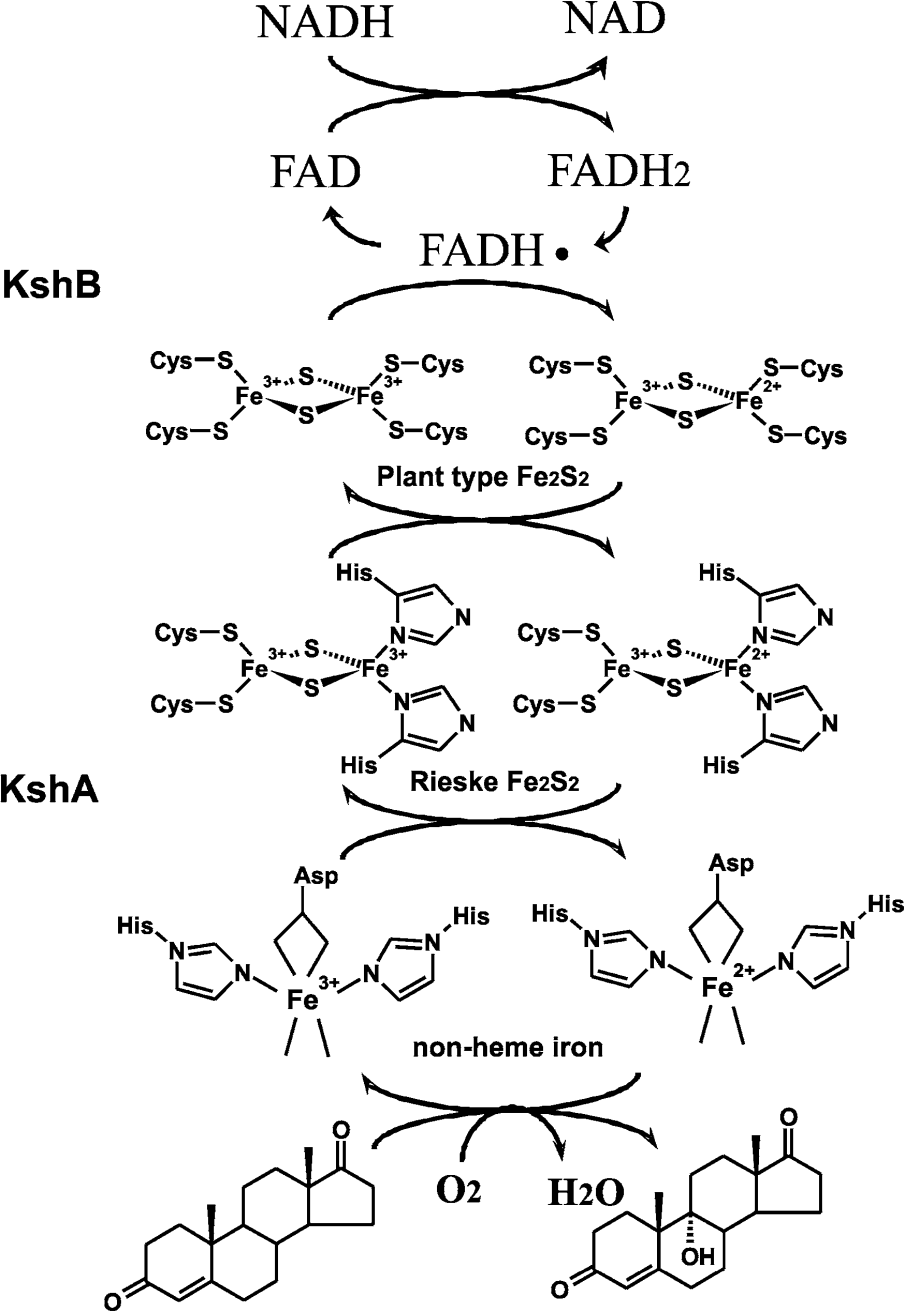
Electron transport chain reactions and substrate hydroxylation catalyzed by KSH. *Arrows* indicate the flow of electrons, starting from the electron donor NADH to the flavin co-factor of KshB, FAD, and via the plant type iron–sulphur cluster, coordinated by four cysteines, of KshB to the Rieske iron–sulphur cluster, coordinated by two cysteines and two histidines, of KshA ending up at the non-heme iron at the core of the catalytic domain. Non-heme iron is coordinated by an aspartate and two histidine residues leaving two binding sites open. Here O_2_ can be bound and one O-atom is used for the hydroxylation of the steroid substrate while the other O-atom is reduced to H_2_O (Petrusma 2011)

**Fig. 3:**
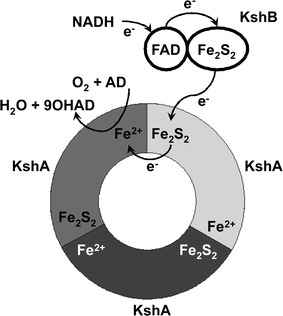
Schematic representation of the typical head-to-tail trimer arrangement of KshA enzymes (adapted from Capyk et al. 2009). Three KshA units are shown in *light grey*, *dark grey* and *black*, forming a circle. Also the FAD co-factor and the iron–sulphur cluster of KshB are depicted. Electrons (e−) are transferred from the iron–sulphur cluster of KshA to the non-heme Fe2+ of the neighbouring KshA unit. AD and 9OHAD indicate 4-androstene-3,17-dione and 9α-hydroxy-4-androstene-3,17-dione, respectively (Petrusma 2011)

Rieske non-heme oxygenases (ROs) constitute a distinct class of enzymes. They are found in a wide range of organisms but occur most abundantly in bacteria. The members of this enzyme family are involved in catabolism of a wide range of substrates, including many aromatic and toxic compounds (Chakraborty et al. 2012). Therefore, these enzymes are of great environmental and industrial importance. ROs are multicomponent redox enzyme systems, consisting of an oxygenase and one or two reducing enzymes. The oxygenase component, which performs the substrate hydroxylation, is an iron–sulphur protein and contains a non-heme iron situated at the active site. KSH enzymes employ an electron transport chain that starts with the oxidation of NADH. The electrons are transferred to the flavin co-factor (FAD) of the ferredoxin reductase component KshB and then transported to the plant type iron–sulphur cluster of KshB. The Rieske iron–sulphur cluster of the KshA oxygenase component subsequently accepts the electrons from KshB. The electrons end up at the non-heme iron situated in the active site of KshA. The mononuclear iron is the site where O_2_ is bound and activated and the substrate is hydroxylated (Batie et al. 1991; Mason and Cammack 1992 (review)) (Fig. 2). The crystal structure of KshA of *M. tuberculosis* revealed that the Rieske iron–sulphur cluster and the non-heme Fe^2+^ catalytic centre are located relatively far away from each other. Crystal structures from other ROs confirm that this is characteristic for this type of protein. However, the typical head-to-tail trimer arrangement positions the Rieske Fe_2_S_2_ in close proximity to the non-heme Fe^2+^ of the neighbouring KshA subunit, enabling transport of electrons between KshA subunits (Fig. 3). A conserved aspartic acid, Asp178 of KshA of *M. tuberculosis* (Capyk et al. 2009), plays a key role in this arrangement by formation of hydrogen bonds, thereby facilitating electron transfer between adjacent subunits (Parales et al. 1999). This aspartate has also been implicated in catalysis (Beharry et al. 2003; Pinto et al. 2006; Tarasev et al. 2006). A common feature of non-heme iron enzymes is the 2-His-1-carboxylate facial triad motif binding the non-heme iron in the core of the active site. The iron is thus coordinated in the protein by three endogenous ligands leaving it largely exposed and creating a very reactive active site (Fig. 2). Up to three exogenous ligands can be bound to the metal centre (Bruijnincx et al. 2008; Hegg and Que 1997 (reviews)). The metal centre of KshA is coordinated at the core of the active site by two histidines (His181 and His186) and an aspartate (Asp304) residue (Capyk et al. 2009). As in several Rieske oxygenases, the iron is bidentate bound to the carboxyl group of the aspartate leaving two sites available for exogenous ligands. This metal centre is more labile compared to the covalently bound heme–iron. However, non-heme iron is a highly catalytic platform able to bind O_2_ and steroid substrate simultaneously in different orientations.

Rieske oxygenases are known to catalyze a range of chemical reactions such as dioxygenation and monooxygenation. Several ROs are known to perform both reactions, e.g. carbazole 1,9a-dioxygenase (Nojiri et al. 1999). Furthermore, ROs are also known to catalyze reactions like desaturation (e.g. Torok et al. 1995), O-dealkylation (Resnick and Gibson 1993), sulfoxidation (Boyd et al. 2004; Lee et al. 1995), desulphonative dioxygenation (Locher et al. 1991) and oxidative carbocyclization (Sydor et al. 2011). This range of reactions performed by ROs demonstrates the large catalytic capacity of the non-heme iron active site. The reaction mechanism of KSH is unknown and although many studies provide insight into the nature of catalysis of ROs, the precise catalytic mechanism of ROs is still unclear. However, some common mechanistic features are apparent for ROs. The first step in catalysis is binding of the substrate in the active site and reduction of the non-heme iron (Fe^2+^). Furthermore, the Rieske iron–sulphur cluster also needs to be in a reduced state. Only when the oxygenase is in this state, O_2_ can bind to the metal centre. This arrangement prevents uncoupling because all components of the reaction are necessary for activation of O_2_ (Barry and Challis 2013; Neidig and Solomon 2005 (reviews)). Crystal structures of naphthalene dioxygenase provided evidence for side-on (η^2^) binding of O_2_ to the metal centre, meaning that both O-atoms are bound to the Fe^2+^. This type of O_2_ binding may explain the high regiospecificity observed for many ROs. This contrasts with CYP enzymes, which bind O_2_ in an end-on fashion, meaning that only one oxygen atom is bound to the heme iron (Karlsson et al. 2003). The versatility and high regiospecificity of ROs makes the enzymes very interesting for industrial applications.

The oxygenase component of ROs is either an α-subunit alone, e.g. KshA, the oxygenase component of KSH, or contains both an α-subunit and a β-subunit (Mason and Cammack 1992 (review)). The β-subunit is smaller and its function has not been elucidated. Several studies indicate a role for the β-subunit in substrate hydroxylation activity (e.g. Jiang et al. 1999). However, other studies indicate that the β-subunit has a structural role and is not involved in substrate specificity or selectivity (e.g. Friemann et al. 2005). 3D structures are available for a range of Rieske non-heme oxygenases. All enzymes are organized in either an α3 trimer, e.g. KshA (Capyk et al. 2009) (Fig. 3) or an α3β3 hexamer, e.g. nitrobenzene dioxygenase (Friemann et al. 2005). An exception is the phthalate dioxygenase of *Burkholderia cepacia* (DB01) which is organized as an α3α3 stacked hexamer (Tarasev et al. 2007). The α-subunit, e.g. KshA, contains a Rieske domain, coordinating the Rieske Fe_2_S_2_ cluster, and a catalytic domain with the typical helix-Grip fold, which is part of the StAR (steroidogenic acute regulatory protein) related lipid transfer (START) domain superfamily. The catalytic domain is composed of a β-sheet flanked by α-helices (Iyer et al. 2001). The oxygenase component of ROs is reduced by a ferredoxin reductase, e.g. KshB in KSH. In three component systems the electrons are shuttled between the oxygenase and the reductase by a small ferredoxin (Mason and Cammack 1992(review)). This ferredoxin has not been detected for KSH.

**Fig. 4:**
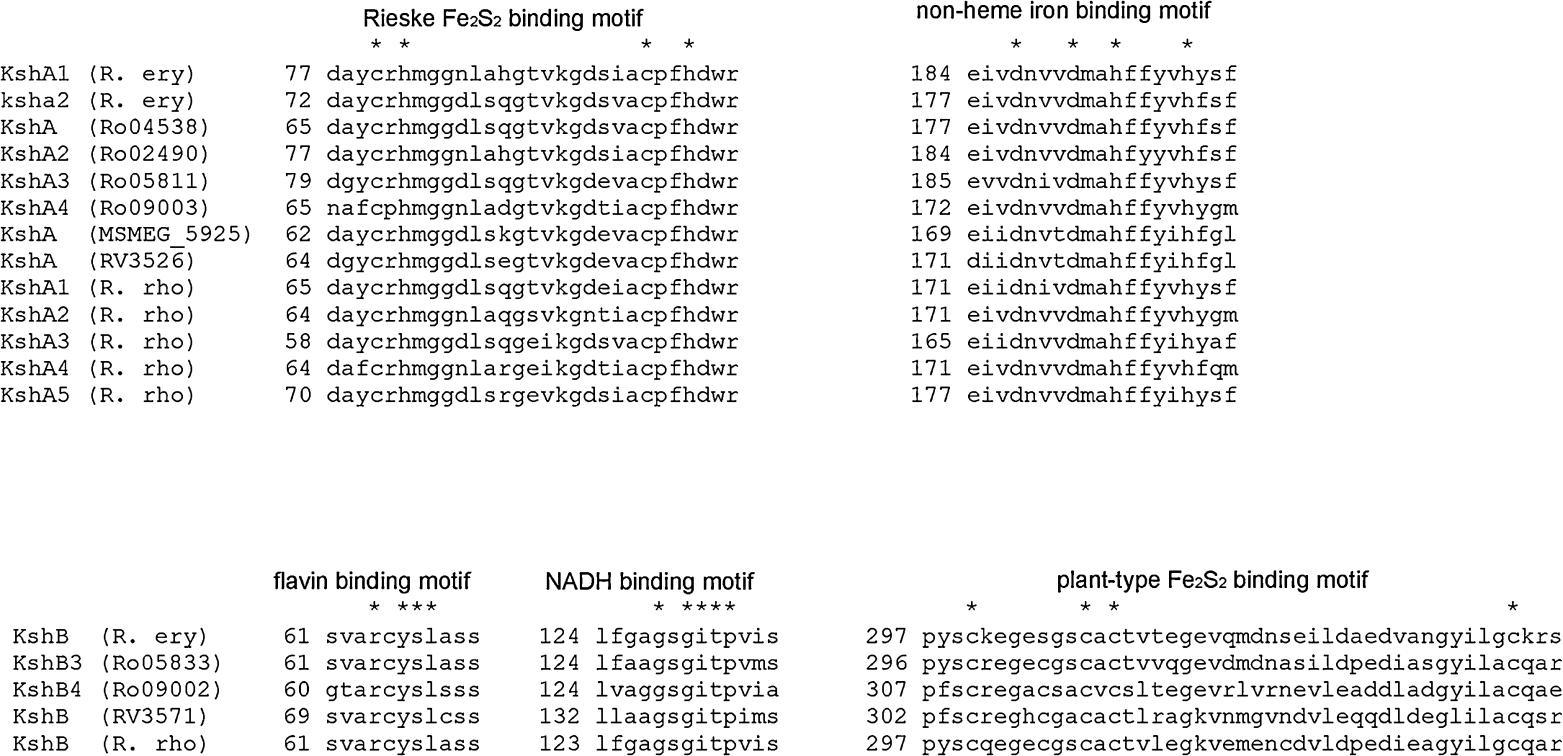
Co-factor binding amino acid sequence motifs in KshA and KshB of *R erythropolis* SQ1 (R. ery) (KshA1, KshB (Van der Geize et al. 2002); KshA2 (Van der Geize et al. 2008), *R. jostii* RHA1 (Ro gene numbering) (Mathieu et al. 2010; Van der Geize et al. 2007). *M. smegmatis* mc^2^155 (MSMEG_5925) (Andor et al. 2006; Arnell et al. 2007), *M. tuberculosis* (RV) (Capyk et al. 2009) and *R. rhodochrous* DSM43269 (R. rho) (Wilbrink et al. 2011). * indicates conserved residues. (Petrusma 2011)

Amino acid sequence analysis of KshA (oxygenase) reveals a Rieske iron–sulphur binding motif and a non-heme Fe^2+^ binding motif, and KshB (reductase) has a flavin, NADH and plant type iron–sulphur binding motifs (Van der Geize et al. 2002) (Fig. 4). KSH was thus classified als a class IA RO enzyme system according to the RO classification system proposed by Batie et al. (1991) (Table 1). This classification is based on the diversity of electron transport components in the oxygenases. The terminal oxygenase is dependent on a ferredoxin reductase for reduction. Several RO systems need a third protein, a small ferredoxin that shuttles electrons between the ferredoxin reductase and the terminal oxygenase. Location, number and nature of the iron–sulphur clusters are used as characteristics to group oxygenases. The Batie classification system consists of three main classes, of which class I and class II are subdivided in group A and B. Class I oxygenases consist of two components, a terminal oxygenase and a ferredoxin reductase. Class IA ferredoxin reductases have N-terminal FMN as co-factor while FAD is co-factor in class IB, located between the iron–sulphur binding domain and the NAD binding domain (Correll et al. 1992). Class II and III oxygenases employ ferredoxin as a third component. In class II systems the ferredoxin reductase contains FAD and the ferredoxin an iron–sulphur cluster. Class II is subdivided in group A and B on the basis of the nature of the iron–sulphur cluster of the ferredoxin. In class III the ferredoxin reductase contains both a FAD and an iron–sulphur cluster. The reductase component of class III shows strong similarity to the reductase of class IB. However, in the class III systems a ferredoxin, containing an iron–sulphur cluster is required for electron transfer between the reductase and the oxygenase (Batie et al. 1991).

**Table 1:** Classification system of Rieske non-heme oxygenases according to Batie et al. (1991)

Class	Number of components	Cofactor of the ferredoxin reductase	Iron–sulphur cluster in ferredoxin	Iron–sulphur cluster in oxygenase
IA	2	FMN [Fe_2_S_2_Cys_4_]	–	[Fe_2_S_2_Cys_2_His_2_] non-heme Fe^2+^
IB	2	FAD [Fe_2_S_2_Cys_4_]	–	[Fe_2_S_2_Cys_2_His_2_] non-heme Fe^2+^
IIA	3	FAD	[Fe_2_S_2_Cys_4_]	[Fe_2_S_2_Cys_2_His_2_] non-heme Fe^2+^
IIB	3	FAD	[Fe_2_S_2_Cys_2_His_2_]	[Fe_2_S_2_Cys_2_His_2_] non-heme Fe^2+^
III	3	FAD [Fe_2_S_2_Cys_4_]	[Fe_2_S_2_Cys_2_His_2_]	[Fe_2_S_2_Cys_2_His_2_] non-heme Fe^2+^

The Batie classification system for ROs has been broadly applied. The biochemical classification has a strong evolutionary basis (Harayama et al. 1992 (review); Nakatsu et al. 1995; Neidle et al. 1991). However with the identification of a growing number of ROs, more and more enzymes are found to have characteristics that do not fit the Batie classification system. KSH was also initially classified as a Class IA RO based on amino acid sequence analysis. However, biochemical characterization of KshB showed the presence of FAD as co-factor indicative for a class IB oxygenase (Capyk et al. 2009; Petrusma et al. 2009).

A new classification system was introduced by Nam et al. (2001) based on the amino acid sequence of the oxygenase components (the alpha subunits). Evolutionary relationships among oxygenases do not appear to be related to substrate specificity, since oxygenases sharing similar substrate ranges can be classified in different groups, based on amino acid sequence similarity. The Nam-system consists of four groups. KSH was classified as a group I RO in this classification system. Group I contains a broad range of mono- and dioxygenases with low amino acid sequence similarity and various protein sizes. The oxygenases are α-monomers. Sequence alignments did not allow elucidation of the specific domains necessary for monooxygenation or dioxygenation specificity. Characteristic for group I oxygenases are 16 or 18 residues between the first histidine residue and the second cysteine residue of the Rieske iron–sulphur binding domain. In contrast, oxygenases of group II, III and IV all have 17 residues separating the first histidine and second cysteine. The two histidines binding the non-heme Fe^2+^ are separated by 3 or 4 amino acids in class I, 4 amino acids in class II and III and 4 or 5 amino acids in class IV. The system proposed by Nam et al. (2001) also allows classification according to the electron transport chain. Group II oxygenases are composed of two components, groups III and IV are made up of three components and group I has either 2 or 3 components. The Batie et al. (1991) and Nam et al. (2001) classification systems are based on the composition of the electron transport chain of oxygenases and on the amino acid sequences of the oxygenases, respectively. Together, they complement each other and give more insight into similarities and differences of ROs. In 2008, another classification system was introduced by Kweon et al. (2008) that incorporated both information about the composition of the electron transport chain as well as the oxygenase component. This classification system is based on information of all components of ROs but is also reliable in classifying incomplete oxygenases systems. Thus, this classification system can predict the presence of unknown electron transport components. The classification consists of five types of oxygenases. KshA was classified as a Type I oxygenase in the Kweon et al. (2008) classification system. A modification of the Kweon classification was suggested by Chakraborty et al. 2012. This modification includes combinations of electron transport chain components based on evolutionary relationships between the different RO components as well as functional properties of the ROs. Oxygenase components of ROs share structural similarities but are very diverse in amino acid sequence. A phylogenetic study, combining structural information and amino acid sequences of the oxygenase components of ROs, suggest that classification of ROs in such broad groups is not feasible (Capyk and Eltis 2012). The RO phylogenetic tree shows two distinct groups but within these groups specific clans cannot be identified. One exception is a large clan in group II, also containing KshA of *M. tuberculosis* H37Rv. Interestingly, KshA is clustered with the eukaryotic Neverland enzyme, a cholesterol 7,8-dehydrogenase. The authors propose a new classification system which is constituted of smaller groups, with functional similarities between the members of such a group. This system would be similar to the naming system of P450s. Since this new classification system is focussed on the oxygenase component of ROs, the system introduced by Batie et al. 1991 would complement the new system because it also includes the other components of the electron transport chain of ROs (Capyk and Eltis 2012).

**Fig. 5:**
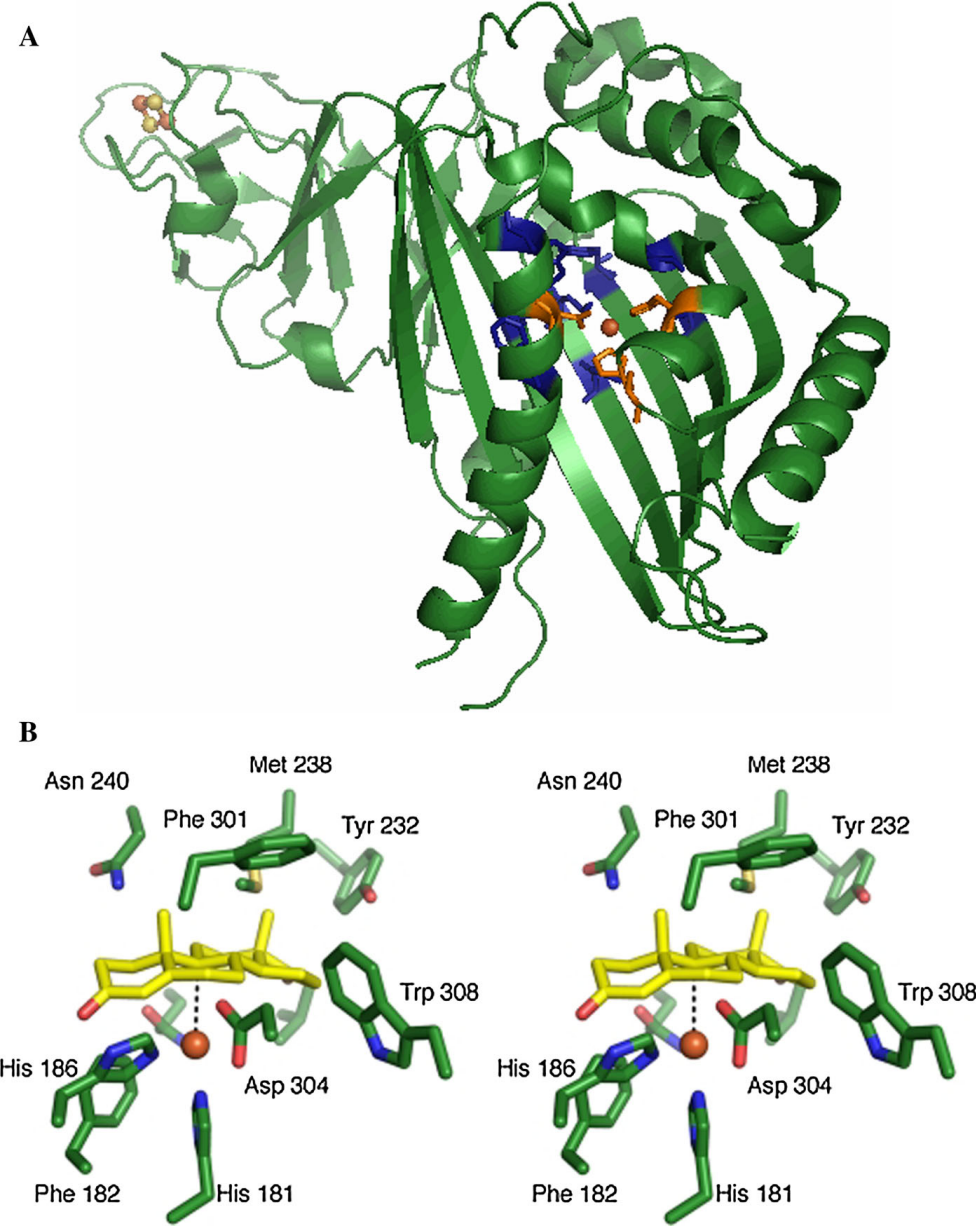
Crystal structure of KshA of *M. tuberculosis* H37Rv (**a**), with non-heme iron coordinating residues in *orange* (His181, His186 and Asp304) and putative substrate interacting residues in *blue* (Val176, Gln204, Tyr232, Met238, Asn240, Asn257, Phe301, Trp308) (adapted from Capyk et al. 2009, PDB: 2ZYL). Stereo image of an ADD docking experiment indicating substrate interacting residues of KshA (**b**) (This figure was originally published in Journal of Biological Chemistry. Capyk et al. (2009) 284:9937–9946 © the American Society for Biochemistry and Molecular Biology.)

After the identification of KSH in *R. erythropolis* SQ1 (van der Geize et al. 2001) a *kshA* gene was also identified in *Mycobacterium smegmatis* mc2 155. *E. coli* cells expressing this KshA displayed C9α-hydroxylating activity on AD and progesterone. Upon purification of the enzyme the hydroxylating activity was greatly reduced (Andor et al. 2006; Arnell et al. 2007). *kshA* and *kshB* of *R. rhodochrous* DSM43269 were co-expressed in *E. coli* and whole cell bioconversion of AD to 9OHAD was observed, with a yield of >60 % after 48 h (Petrusma et al. 2009). KshA and KshB of both *R. rhodochrous* DSM43269 (Petrusma et al. 2009) and *M. tuberculosis* (Capyk et al. 2009) has been purified in active forms and were dependent on NADH as electron donor for activity. KshA of *M. tuberculosis* was purified anaerobically (Capyk et al. 2009) while KshA of *R. rhodochrous* DSM43269 was purified aerobically but only remained in an active form when purified together with KshB, resulting in an active KSH enzyme (Petrusma et al. 2009). The 3D structure of KshA of *M. tuberculosis* has been elucidated (Capyk et al. 2009). It contains a Rieske iron–sulphur cluster and a divalent non-heme iron located in the core of the active site. KshA subunits are organized in trimers, forming a typical α3-fold as mentioned above (Fig. 3). The catalytic domain of KshA is organized in the typical Helix–Grip fold (see above) but differs from other known α3-ROs in the shape of the substrate-binding pocket and position of active site channel, equipped for steroid substrates. KshA has distinct C-terminal features involved in formation of the α-trimer. It was suggested that KshA is an archetypical RO because the structure shows a minimal catalytic domain compared to other available RO structures (Capyk et al. 2009) (Fig. 5). In vitro bioassays with KSHs of *M. tuberculosis* H37Rv and *R. rhodochrous* DSM43269 showed that the enzymes can use 3-ketosteroids as substrates. They can tolerate different configurations of the A-ring and some enzymes display hydroxylation activity on steroids with longer C17 side chains. As mentioned above, KSH of *M. tuberculosis* shows high preference for the CoA thioester intermediate of cholesterol side chain degradation compared to the tested C17-ketosteroids. These studies indicate that KSH activity can occur at different stages of the sterol degradation pathway (Capyk et al. 2011; Petrusma et al. 2011).

## Physiological roles of KSH enzymes

Sterols and steroids can be degraded by a range of microorganisms, many of which are actinobacteria, e.g. *Rhodococcus* and *Mycobacterium* species, and serve as carbon- and energy sources for growth (Donova 2007; Fernandes et al. 2003; Malaviya and Gomes 2008; Van der Geize and Dijkhuizen 2004 (reviews)). KSH is a key enzyme in bacterial sterol catabolic pathways. The essential role of KSH in opening of the steroids polycyclic ring structure was demonstrated for the first time in *R. erythropolis* SQ1. The *kshA* and *kshB* gene deletion mutants were able to grow on 9-OHAD but not on AD. This strain, however, was still able to grow on phytosterols, indicating the presence of additional KshA enzymes or an alternative degradation pathway (Van der Geize et al. 2002). A *kshA* disruption mutant of *Mycobacterium smegmatis* mc2 155 incubated with sitosterol accumulated AD and ADD (Andor et al. 2006). *R. rhodochrous* DSM43269 expresses 5 KshA homologs. A *kshA* null mutant was constructed by gene deletion mutagenesis (strain RG32) to fully block opening of the steroids polycyclic ring structure of cholesterol and β-sitosterol; this resulted in accumulation of ADD and 3-oxo-23,24-bisnorchola-1,4-dien-22-oic acid (1,4-BNC) (Wilbrink et al. 2011). These mutants still degraded the sterol side chain, thus demonstrating the potential of mutant bacterial strains to convert relatively cheap sterol substrates into valuable steroids.

Rhodococci were found to have relatively large genomes, displaying strong gene diversity. Multiple copies are present for many genes, encoding (iso)enzymes for specific steps in catabolic pathways. Such a multiplicity was also observed for *kshA*. The genome of *R. erythropolis* SQ1 harbours at least 3 *kshA* genes (Van der Geize et al. 2008). *R. jostii* RHA1 encodes four sterol catabolic gene clusters, each of which contains a *kshA* gene, namely *ro04538* (*kshA*), *ro02490* (*kshA2*), *ro05811* (*kshA3*) and *ro09003* (*kshA4*) (Mathieu et al. 2010; McLeod et al. 2006; Van der Geize et al. 2007). A total of five *kshA* homologues were identified in *R. rhodochrous* DSM43269 (Wilbrink et al. 2011) and *Mycobacterium* sp. VKM Ac-1817D (Bragin et al. 2013), and seven *kshA* homologues are present in the genome of the pathogenic bacterium *R. equi*. Multiplicity for *kshB* is not as excessive as for *kshA*. Apparently, KshB is able to serve as a reductase for several KshA enzymes. *In vitro* bioassays of the five KshA homologues of *R. rhodochrous* DSM43269 also shows that all KshAs are able to use the same KshB as reductase (Petrusma et al. 2011). Little is known about the physiological roles of the different KshA homologues. *In vivo*, each of these KshA homologues may have a specific role in the host bacterium, degrading certain sterol/steroid substrates. Sterol catabolic gene cluster 1 of *R. jostii* RHA1 was designated as a cholesterol degradation gene cluster. Most genes within this cluster are upregulated during growth on cholesterol, including *kshA* (ro04538). The study showed that several pathogenic bacteria also harbour a cholesterol degradation cluster and in *Mycobacterium bovis* bacillus Calmette–Guérin *kshA* was found to be upregulated during growth on cholesterol. A cholesterol catabolic gene cluster was also identified in *M. tuberculosis* (Van der Geize et al. 2007).

**Fig. 6:**
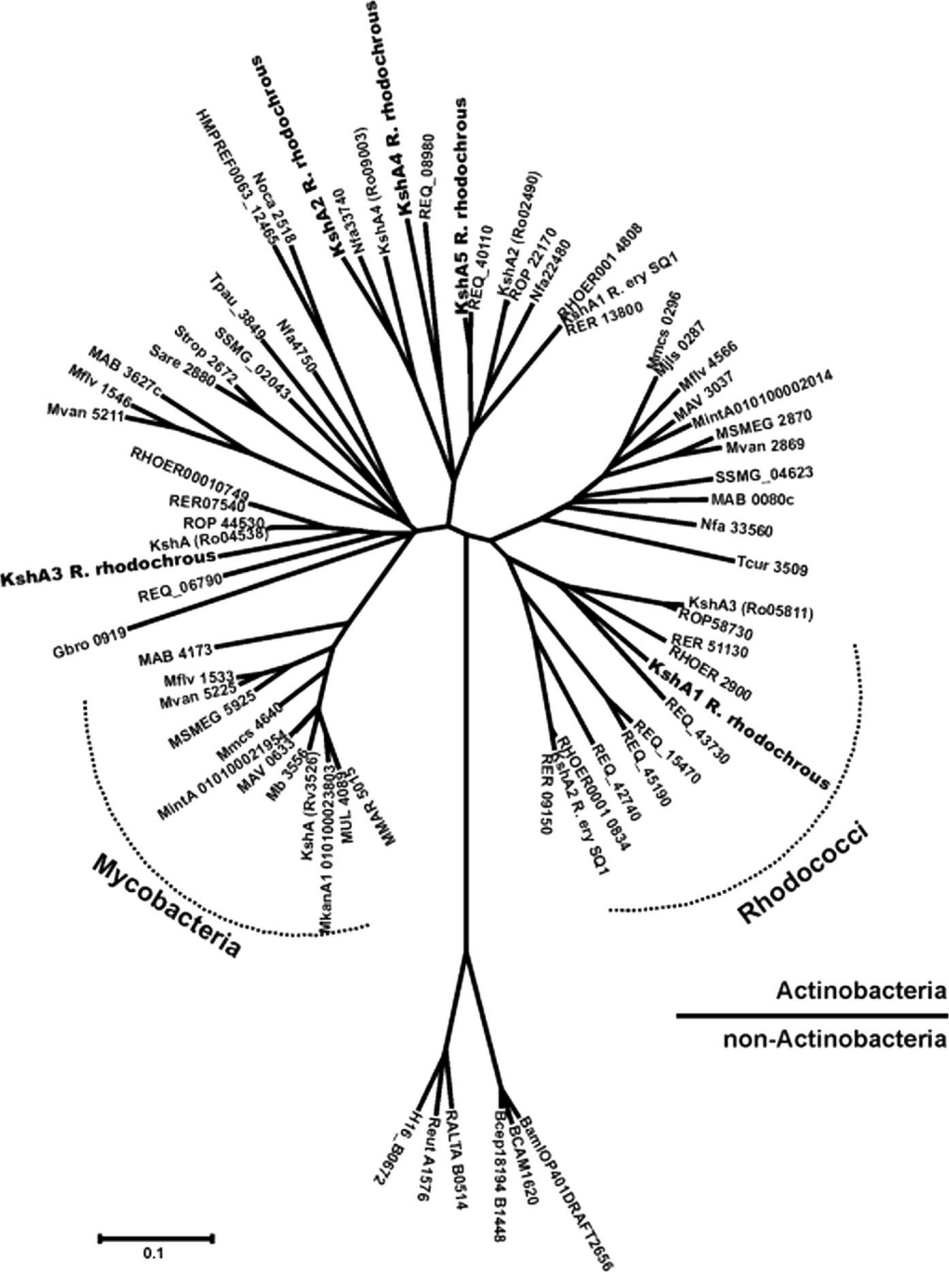
Phylogenetic tree of bacterial KshA enzymes (Petrusma et al. 2011)

*R. jostii* RHA1 gene expression has been analyzed during growth on 7-ketocholesterol (7-KC) and revealed that a set of genes, among which *kshA3*, was specifically upregulated during growth on 7-KC compared to cholesterol. However, many genes were upregulated on both sterols suggesting a common degradation pathway (Mathieu et al. 2010). Promoter activity experiments with *kshA2* of *R. erythropolis* SQ1 showed that *kshA2* is highly induced by 9α-hydroxy-4-androstene-3,17-dione (9-OHAD), a KSH product. It was speculated that this *kshA* homologue prevents accumulation of ADD to toxic levels during sterol catabolism (Van der Geize et al. 2008). Molecular studies with the five KshA homologues of *R. rhodochrous* DSM43269 indicated that these isoenzymes are involved in degradation of specific types of sterols and/or at different levels in the degradation cascade. KshA1 of *R. rhodochrous* DSM43269 appears to be dedicated to cholate catabolism. Expression of *kshA1* was induced during growth on cholic acid. Furthermore, the growth of a *R. rhodochrous* DSM43269 *kshA* null mutant, unable to grow on cholate, was restored to wild type level by complementation by KshA1, and the KshA1 enzyme also showed substrate preference for an intermediate in cholate catabolism (Petrusma et al. 2011). KshA1 of *R. rhodochrous* and KshA3 of *R. jostii* RHA1 are clustered in the same group in a KshA phylogenetic tree (Fig. 6). Phylogenetic analysis showed clustering of KshA enzymes in different groups. It was hypothesized that the members of such a group are functionally related, involved in catabolism of specific steroids (Petrusma et al. 2011). The gene cluster of *R. jostii* RHA1 harboring *kshA3* (*ro05811*) was found to be essential for cholate catabolism. Many genes in the cluster, among which *kshA3*, were found to be highly upregulated during growth on cholate compared to cholesterol. Several bacteria harbouring orthologous gene clusters in their genomes were also found to be able to grow on cholate while all tested bacteria lacking such a gene cluster were unable to grow on this bile acid. A *kshA3* deletion mutant confirmed that the KshA3 enzyme is essential for growth on cholate (Mohn et al. 2012).

## KSH is essential for the pathogenicity of *M. tuberculosis*

*M. tuberculosis* has a much smaller genome than most *Rhodococcus* species characterized and does not display the high gene multiplicity characteristic for most rhodococci. Indeed, the genome of *M. tuberculosis* harbours one *kshA* gene and one *kshB* gene. *M. tuberculosis* is one of the most notorious human pathogenic bacteria. Each year approximately 1.5 million deaths are caused by the disease tuberculosis and millions of people are infected (www.who.int/tb/country/en/index.html). *M. tuberculosis* is such a successful pathogen because it is able to survive for long periods of time (chronic) within macrophages in the lungs (Meena and Rajni 2010 (review)). In recent years it was found that cholesterol catabolism plays an important role in the survival of *M. tuberculosis* in macrophages (Rengarajan et al. 2005; Sassetti and Rubin 2003; Schnappinger et al. 2003; Van der Geize et al. 2007). The precise role of cholesterol degradation in *M. tuberculosis* however is not clear. Several enzymes were found to be essential for survival of *M. tuberculosis* during infection (see below), but cholesterol does not serve as the sole energy source during infection (Yang et al. 2011). It has been suggested that steroid intermediates of the bacterial cholesterol degradation pathway modulate the immune response of the human host (Klink et al. 2013; Yam et al. 2011 (review)).

Several studies have shown that cholesterol catabolism in *M. tuberculosis* is essential in the chronic phase of infection and in IFN-γ-activated macrophages but not during the onset of infection or in resting macrophages (Nesbitt et al. 2010; Pandey and Sassetti 2008). However, transposon mutagenesis studies (Rengarajan et al. 2005; Sassetti and Rubin 2003) and targeted gene inactivation studies of genes coding for enzymes involved in steroid ring degradation (Hu et al. 2010; Yam et al. 2009), indicated that sterol catabolism is essential in the early stages of infection as well as in the chronic phase. Both KSH components were found to be essential for pathogenesis. The separate *kshA* and *kshB* deletion mutants were unable to survive in mice models, and in macrophages, in the early stage of infection as well as in the chronic phase. These mutant strains, unlike wild type *M. tuberculosis*, were unable to catabolize cholesterol, AD and 5α-androstane-3,17-dione (5α-AD). The *kshB* deletion mutant was also impaired in biosynthesis of penta-acylated trehalose (PAT), a glycolipid located at the surface of the cell wall. The data suggests that KshB can serve as a reductase for different oxygenases (Hu et al. 2010).

## Engineering of Rieske non-heme oxygenase enzymes

The essential role of KSH in the pathogenicity of *M. tuberculosis* as well as its potential in production of pharmaceutically interesting steroids make these enzymes an interesting target for protein engineering. One of the best studied ROs is biphenyl dioxygenase. Mutational engineering of this class III oxygenase (Batie et al. 1991) has resulted in enhanced activities, changed and/or expanded substrate ranges and altered regio selectivity (Furukawa et al. 2004 (review)). This susceptibility for enzyme engineering of biphenyl dioxygenase is in agreement with the finding that enzyme homologues from a *Burkholderia* strain and a *Pseudomonas* strain only differ by 20 amino acids, of a total of ~ 460 amino acid residues, while showing clear differences in functionality. Indeed, recombination of the genes encoding these highly similar enzymes by DNA shuffling resulted in altered substrate ranges and enhanced activities (Kumamaru et al. 1998). Intriguingly, the 5 KshA homologues of *R. rhodochrous* DSM43269 (Petrusma et al. 2011), as well as the KshA of *M. tuberculosis* (Capyk et al. 2009), all perform C9α-hydroxylation of steroid molecules while sharing ~60 % amino acid identity. However, all amino acid residues predicted to interact with the polycyclic ring structure of the substrate bound in the active site (Fig. 5) are conserved among known KshAs (Capyk et al. 2009). The KshA_DSM43269_ homologues were all found to act on 3-ketosteroids showing overlap in substrate ranges, but with differences in substrate preference (Petrusma et al. 2011).

Naphthalene 1,2-dioxygenase of *Pseudomonas* sp. strain NCIB 9816-4 also has been well studied and subjected to several mutagenesis studies. This RO uses a large range (> 60) of aromatic compounds as substrates and performs different types of reactions, i.e. di- and monooxygenation, desaturation, sulphoxidation. The suitability of this RO for enzyme engineering was demonstrated by the fact that several point mutations around the active site affected substrate specificity as well as regio- and enantioselectivity (Ferraro et al. 2006; Parales 2003 (review)). The amino acid sequence identity between KshA, biphenyl dioxygenase and naphthalene 1,2-dioxygenase however is very low. Carbazole 1,9α-dioxygenase (CARDO, *Janthinobacterium* sp. strain J3) shares about 18 % amino acid sequence identity with KshAs of both *R. rhodochrous* DSM43269 and *M. tuberculosis* and this RO is classified as a class III oxygenase on the basis of the electron transport chain enzymes (Nojiri et al. 2005). Point mutations of residues lining the substrate binding pocket resulted in CARDO mutants displaying changes in substrate specificity. Most of these mutated residues are situated near the active site. However, mutation of a residue near the entrance of the substrate binding pocket, close to a loop situated at the entrance of the substrate binding pocket, was found to improve activity on specific substrates (Uchimura et al. 2008). Similar loop regions are situated at the entrance of the substrate binding domain of many ROs. It has been speculated that this feature is involved in accommodation of substrates in the active site (Ferraro et al. 2005 (review)). Indeed, a study on chimeric KshA enzymes of *R. rhodochrous* DSM43269, with exchange of regions of the catalytic domain of two KshA homologues with different substrate preferences showed that the loop region, located at the entrance of the active site, strongly influences substrate specificity (Petrusma et al. 2012). A docking experiment with KshA of *M. tuberculosis* and 3-oxo-23,24-bisnorchola-1,4-dien-22-oyl-coenzyme A thioester (1,4-BNC-CoA) as substrate indicated the importance of amino acid residues in a pocket located at the entrance of the active site, which is the predicted location of the CoA group of this preferred substrate. In contrast to the amino acid residue at the active site that interacts with the polycyclic ring structure of ADD (Capyk et al. 2009), the residues near the entrance of the active site interacting with the CoA group are not conserved among known KshAs, indicative for differences in substrate range and specificity of the different KshAs (Capyk et al. 2011).

Detailed insights into the structure and function relationships of KshA, and the amino acid residues involved in substrate range and specificity, as well as regioselectivity of the hydroxylation, will provide a firm basis for subsequent rational engineering of KshA enzymes to improve synthesis of desired bioactive steroids. Also, since KSH was found to be essential for pathogenicity of *M. tuberculosis* (Hu et al. 2010) such insights may allow structure-based design of inhibitors against KSH activity to combat tuberculosis.

## Conclusions

Knowledge on KSH has increased substantially in recent years, paving the way for biotechnological exploitation of their catabolic potential. KSH is a key enzyme in bacterial steroid catabolism and therefore of strong physiological importance for a wide range of sterol degrading bacteria. Mutant bacterial strains devoid of KSH activity may convert relatively cheap sterols into bioactive steroids that are pharmaceutically interesting. KSH enzymes also are essential for pathogenicity of the notorious pathogen *M. tuberculosis*. Knowledge about the KSH catalysed reaction may allow design of specific enzyme inhibitors to combat tuberculosis. In summary, KSH enzymes are highly interesting for many biotechnological and medical applications. Further research will focus on (a) the regulation of their expression in bacterial hosts, against the background of other enzymes involved in sterol conversion, aiming to increase efficiency of metabolic conversions, (b) enzyme structural elements determining substrate/product specificity, followed by enzyme engineering to improve enzymatic conversions.
